# BI-RADS 3: Current and Future Use of Probably Benign

**DOI:** 10.1007/s40134-018-0266-8

**Published:** 2018-01-27

**Authors:** Karen A. Lee, Nishi Talati, Rebecca Oudsema, Sharon Steinberger, Laurie R. Margolies

**Affiliations:** 1 Department of Radiology, The Mount Sinai Medical Center, 1176 Fifth Avenue, Box 1234, New York, NY 10029 USA; 2 Department of Radiology, The Icahn School of Medicine at Mount Sinai, New York, NY USA

**Keywords:** BI-RADS 3, Probably benign, Breast imaging reporting and data system, MRI, Mammography, Breast ultrasound, Breast cancer screening

## Abstract

**Purpose of Review:**

Probably benign (BI-RADS 3) causes confusion for interpreting physicians and referring physicians and can induce significant patient anxiety. The best uses and evidence for using this assessment category in mammography, breast ultrasound, and breast MRI will be reviewed; the reader will have a better understanding of how and when to use BI-RADS 3.

**Recent Findings:**

Interobserver variability in the use of BI-RADS 3 has been documented. The 5th edition of the BI-RADS atlas details the appropriate use of BI-RADS 3 for diagnostic mammography, ultrasound, and MRI, and discourages its use in screening mammography. Data mining, elastography, and diffusion weighted MRI have been evaluated to maximize the accuracy of BI-RADS 3.

**Summary:**

BI-RADS 3 is an evolving assessment category. When used properly, it reduces the number of benign biopsies while allowing the breast imager to maintain a high sensitivity for the detection of early stage breast cancer.

## Introduction

Efforts to improve the specificity and cost-effectiveness of screening mammography led to the development and widespread acceptance of short-term follow-up of probably benign findings. The purpose of the short-term follow-up algorithm is to reduce false-positive findings while retaining a high sensitivity for early stage breast cancer [1]. Probably benign (BI-RADS 3) has been formally established as a unique assessment category in the BI-RADS Atlas [2]. Designating a finding as probably benign in mammography is meant to indicate that the finding has a 2% or less chance of malignancy [3]. In practice, 0.9–7.9% of probably benign mammographic findings are upgraded to suspicious and proceed to biopsy [1, 4–6].

BI-RADS 3 is perhaps the most difficult of the assessment categories for the breast imager to properly use. Indeed, Michaels et al. have shown that there is considerable interobserver variability in the assessments of mammographic Bi-RADS 3 findings [7] and Grimm et al. have shown the same for MRI [8]. Ortiz-Perez has shown that formal instruction in the ultrasound BI-RADS lexicon improves the characterization of findings and BI-RADS assessments [9]. BI-RADS 1 and 2—normal and benign—as well as BI-RADS 4 and 5—suspicious or highly suspicious—are relatively straightforward. BI-RADS 3 lurks, however, in the middle and has significantly different meanings for mammography, ultrasound, and MRI and indeed is audited differently for the three modalities. BI-RADS 3 creates a wide variety of actions and reactions. It causes patient anxiety, eliminates some unneeded biopsies, and is often ignored by patients and referring clinicians. Radiologists who are not sure what to do with a finding often overuse BI-RADS 3. This paper will discuss the appropriate use of BI-RADS 3 with 3 core principles: (1) if a lesion is indeterminate or has worrisome features it is not BI-RADS 3; (2) BI-RADS 3 should not be used to delay diagnosis of a malignant appearing finding; (3) BI-RADS 3 should only be used after a full diagnostic workup.

The typical follow-up protocol for all modalities is similar. For mammography, for example, this includes the assignment of BI-RADS 3 at diagnostic imaging. At 6 months from the screening exam that prompted the recall, another diagnostic evaluation is completed and the finding is biopsied if warranted. Assuming the finding is stable, it is again assigned BI-RADS 3 and a bilateral mammogram in 6 months is performed. At 12 months from the screening exam, the diagnostic mammogram is repeated and is generally again assessed as BI-RADS 3 unless upgraded to BI-RADS 4 or 5. At the 12-month mark, although the exam can be BI-RADS 3, the follow-up interval can be increased to 1 year. Assuming 24 months of stability, the patient can revert to BI-RADS 2 or one can continue as BI-RADS 3 recommending imaging in 1 year assuming no need for biopsy. A finding can be upgraded to BI-RADS 4 or 5 or downgraded to BI-RADS 2 at any point along the follow-up. The timing of follow-up exams is the same for ultrasound and MRI.

Compliance with BI-RADS 3 recommendations is far from perfect. A recent study by Chung et al. found that 83.3% complied with the first 6-month follow-up, decreasing over time to 75.9% at 12 months and 53.9% at 24 months [10]. A strong navigation program is needed to maximize compliance, but even with one’s best efforts patients may not return for reasons beyond the radiologist’s control including insurance issues especially for MRI follow-ups.

## BI-RADS 3 in Mammography

**Fig. 1:**
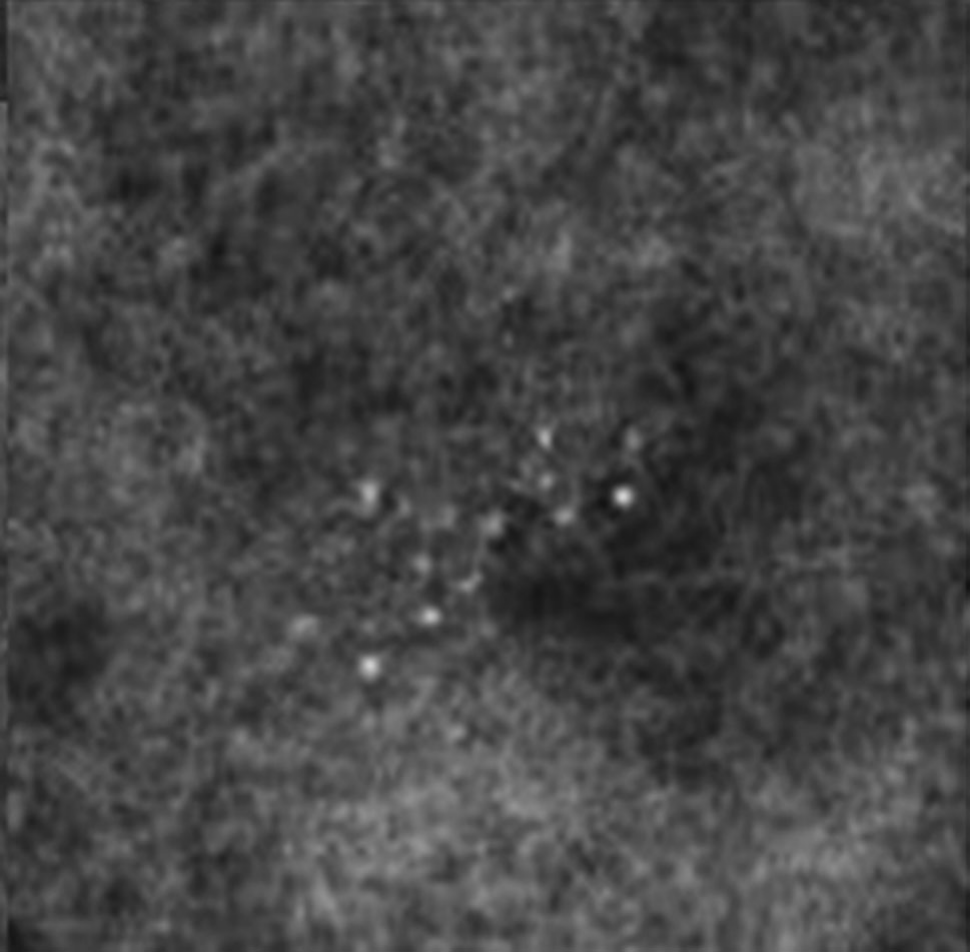
Mammographic appearance of solitary group of round or punctate calcifications, which are appropriate for BI-RADS 3

**Fig. 2:**
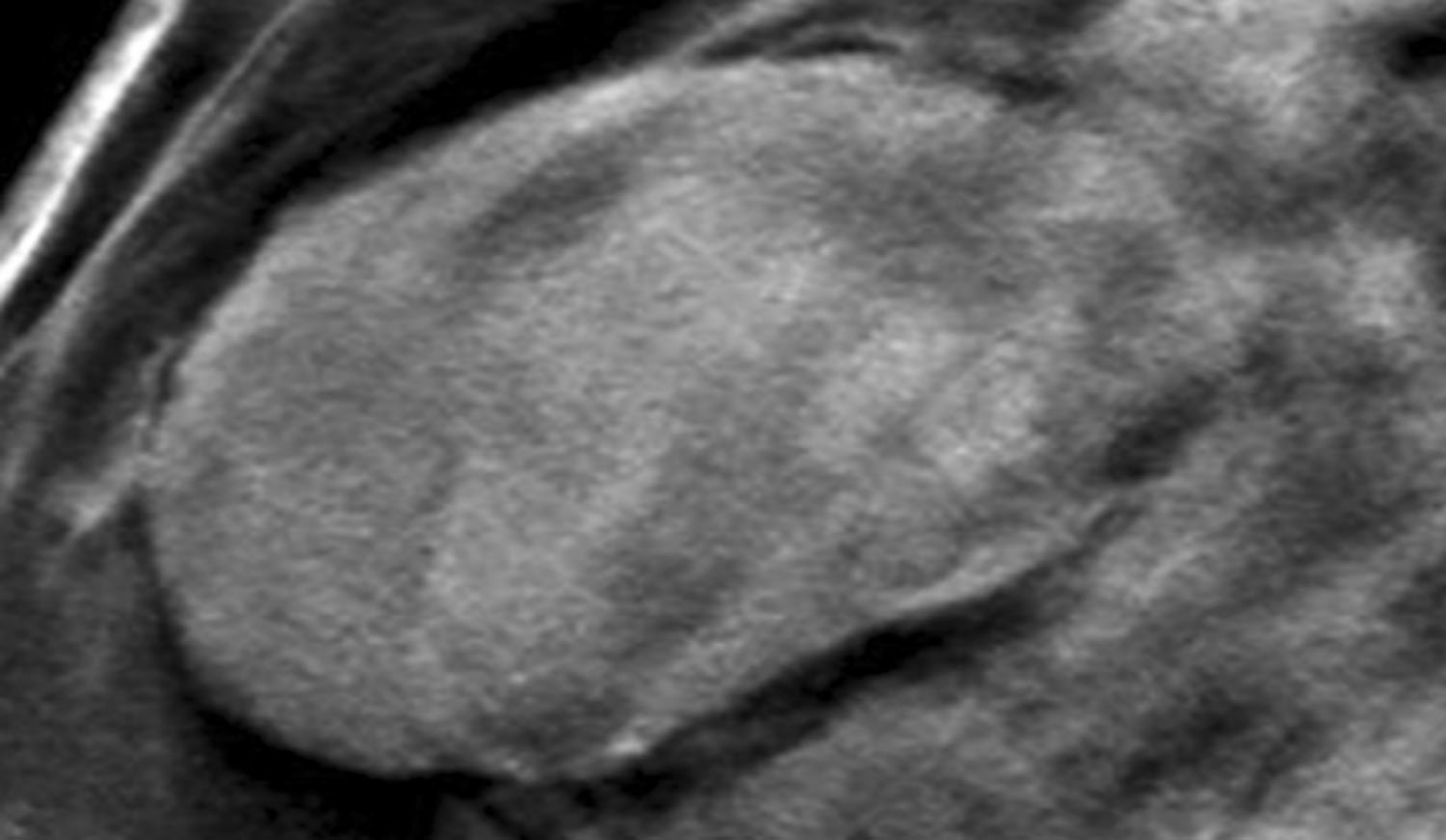
Non-calcified circumscribed oval mass on mammography

**Fig. 3:**
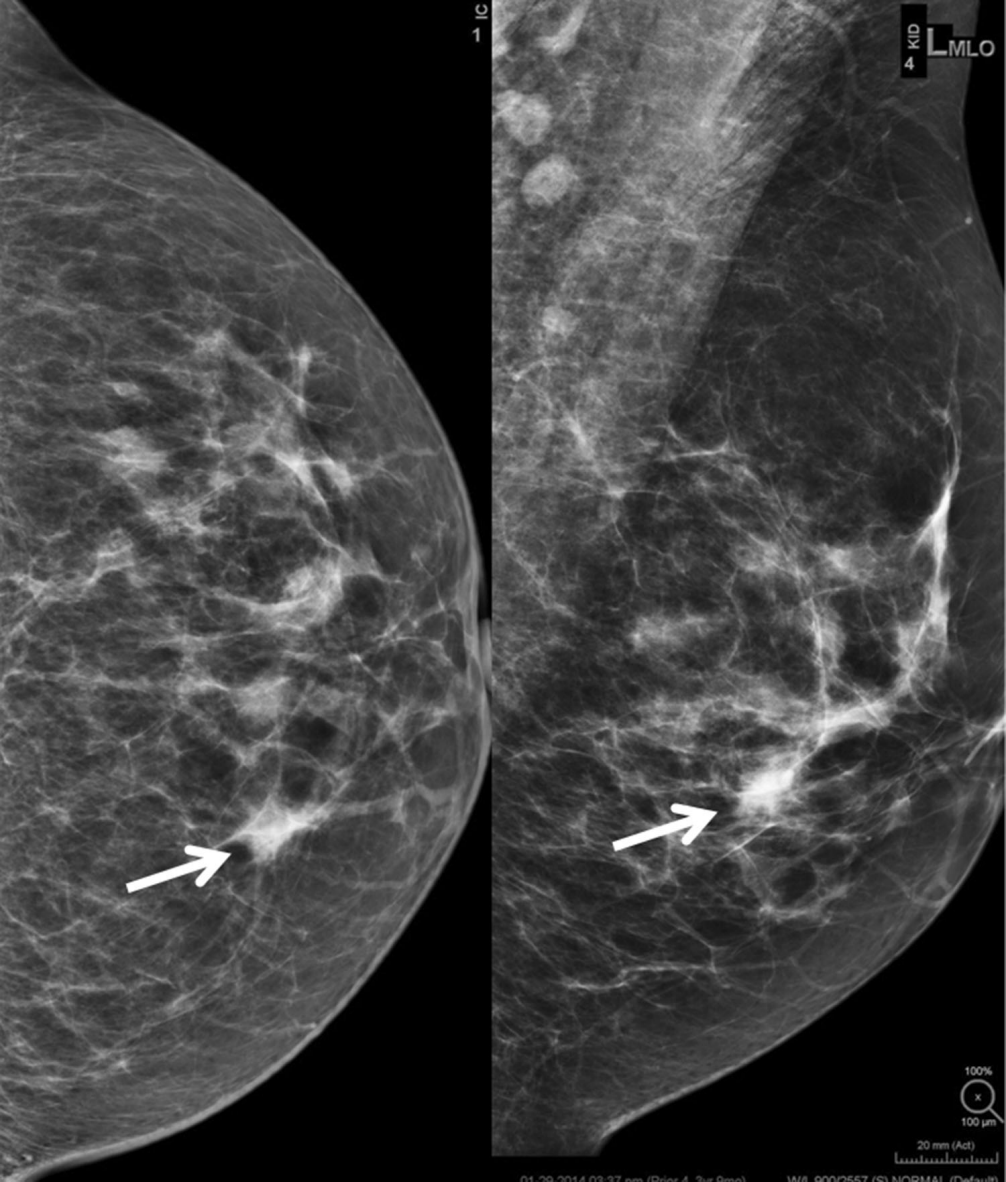
Focal asymmetry without calcifications or architectural distortion

BI-RADS 3 is not appropriate at screening mammography. After a complete diagnostic evaluation, classifying a mammographic finding as a BI-RADS 3 is highly predictive of benignity [4, 11, 12] and allows for short interval follow-up rather than biopsy. While a BI-RADS 3 categorization allows for a decrease in the number of biopsies and their associated risks and costs, it should only be used to describe specific findings including a solitary group of round or punctate calcifications (Fig. 1), a non-calcified well-circumscribed solitary mass (Fig. 2), or a focal asymmetry (Fig. 3) without calcification or architectural distortion. [1, 13–15].

**Fig. 4:**
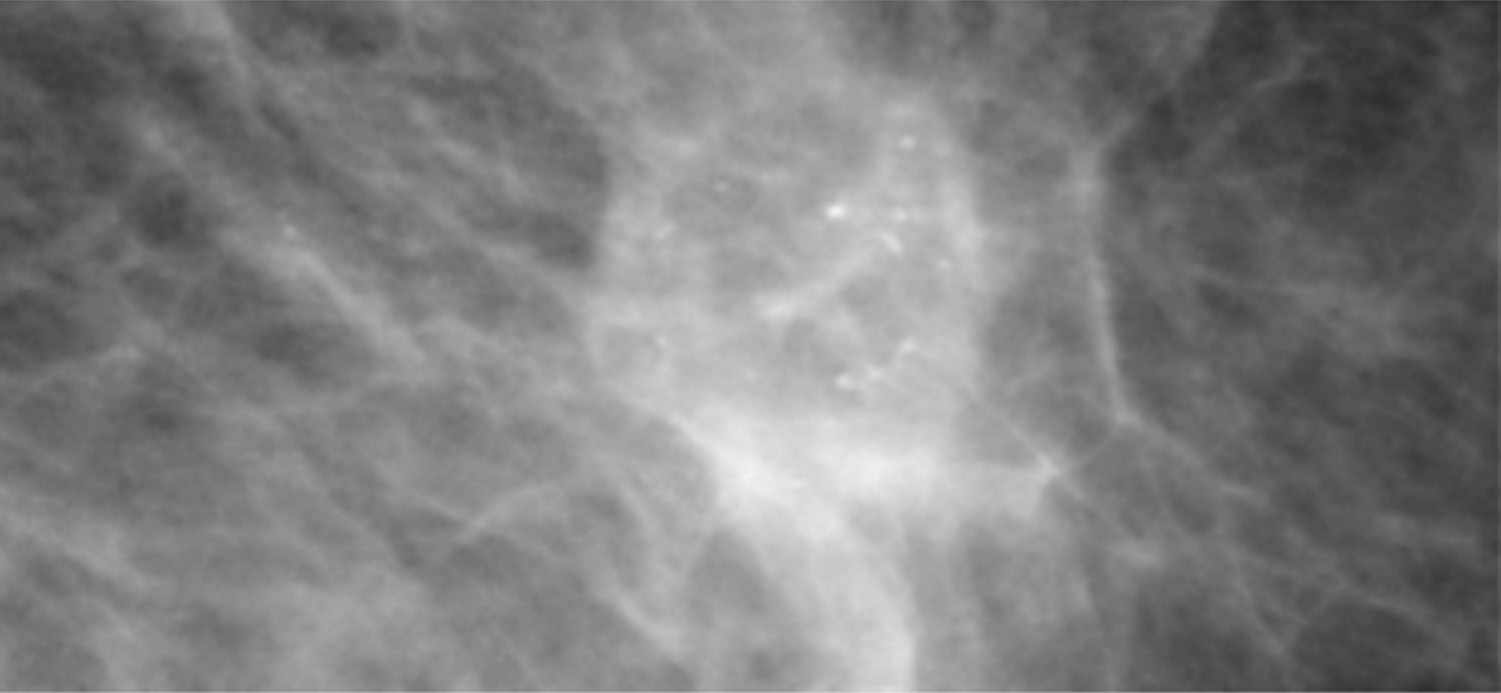
Calcifications in a patient with a history of trauma, consistent with fat necrosis

A single group of punctate or round calcifications may be classified as a BI-RADS 3 after appropriate evaluation with magnification views [1]. Additionally, calcifications suggestive of early fat necrosis (Fig. 4) in a patient who has undergone biopsy or trauma as well as calcifications that the radiologist believes are most likely vascular can be categorized as probably benign [13]. During follow-up, an increase in the number of calcifications that is not consistent with an evolving benign cause, or a change in calcification morphology causing them to appear more suspicious should prompt a biopsy recommendation [16]. For BI-RADS 3 to be properly used, the calcifications must be properly assessed. Amorphous calcifications, for example, carry a greater risk of malignancy and should not be assigned BI-RADS 3 [17].

A non-calcified solid mass that is round or oval with circumscribed margins can be classified as BI-RADS 3 [15]. If a mass has overlapping fibroglandular tissue obscuring the margins, it can be categorized as probably benign if at least 75% of the margins are circumscribed and no portion of the visualized margin is suspicious. This may require obtaining spot compression or magnification views [15, 18] or an ultrasound for further evaluation [19, 20]. If a mass in this category demonstrates stability, it can be categorized as definitively benign and assessed as BI-RADS 2 [5, 6]. However, a mass with benign characteristics that demonstrates interval growth or a suspicious change in morphology cannot appropriately be considered a BI-RADS 3 and should be recommended for biopsy [21, 22••].

A focal asymmetry is a density with concave borders, which is contained in a single quadrant and is seen on at least two mammographic projections [2]. It is often interspersed with fat and in the absence of calcification or architectural distortion it can be classified as probably benign if initially detected on a baseline examination [1]. However, if a focal asymmetry is new or increased in size it is not a true focal asymmetry, but rather a developing asymmetry and should be biopsied as developing asymmetries seen at screening have a > 12% chance of malignancy [23].

**Fig. 5:**
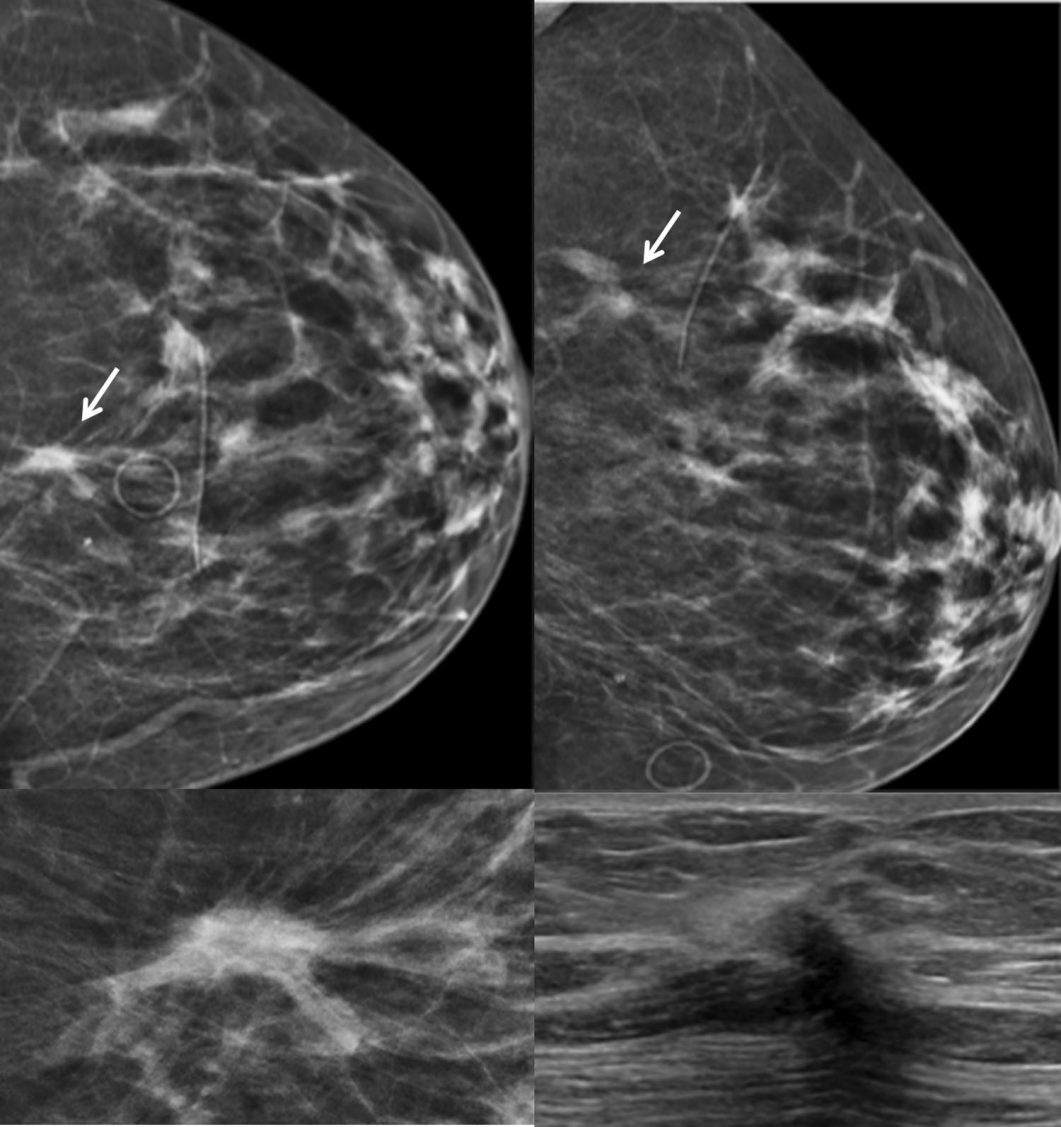
Focal asymmetry with post-surgical architectural distortion seen on mammography and ultrasound in a patient with prior breast surgery. This was initially assessed as BI-RADS 3 but subsequent follow-up mammograms demonstrate long-term stability

The BI-RADS atlas also provides some room for radiologist’s discretion by allowing one to place findings in a BI-RADS 3 category if one’s personal experience would allow one to justify the assessment. For example, in addition to calcifications that may be vascular or fat necrosis, an asymmetry or distortion thought to represent a post-surgical scar may be assigned BI-RADS 3 (Fig. 5). Also, if there are technical differences between exams that make it difficult to assess stability, one could assess a finding as BI-RADS 3. This happened with the change from analog to digital and now happens with the change from full field digital to digital breast tomosynthesis (DBT) [2].

The BI-RADS 3 assessment should not be assigned at screening mammography; one reason is the relatively high rates of non-compliance with short interval follow-up recommendations [24, 25]. Omitting diagnostic workup could delay diagnosis of cancer at a lower stage and smaller size [26], potentially impacting treatment and/or prognosis. In contrast, unnecessary follow-up of a finding that could have been proven benign at diagnostic workup can increase overall cost and patient anxiety. For example, prompt workup of a mass seen on screening mammography may indicate that it is a benign cyst, and patient would not need short interval follow-up examinations.

Digital breast tomosynthesis (DBT) has impacted the use of BI-RADS 3 at diagnostic mammography. Raghu et al. found that over a 3-year time frame the use of BI-RADS 3 at diagnostic mammography fell from 33.3 to 16.4% with no change in the percentage of BI-RADS 4 and 5 findings. Many of those who were previously placed into the probably benign assessment category are now given a normal report. This is in large part due to DBT’s ability to assess focal asymmetries as normal tissue [27]. Similarly, McDonald et al. found that screening with DBT decreased the overall number of patients recommended for short interval follow-up by a mean of 2.4 women per 1000, compared to screening with digital mammography [28••].

While the use of BI-RADS 3 in mammography continues to evolve, it has served as a paradigm for its implementation in ultrasound and MRI.

## Ultrasound BI-RADS 3

Ultrasound is readily available, uses no ionizing radiation, and is well tolerated by patients. In women with dense breast tissue, supplemental breast ultrasound imaging can increase cancer detection rates by 2.3–4.6 per 1000. However, in finding more cancers, supplemental ultrasound will also discover more benign masses that are not characteristically benign in appearance, increasing the number of biopsies and false-positive rates [29–34, 35•, 36, 37].

BI-RADS ultrasound descriptors have been shown to distinguish between malignant and benign masses with high positive and negative predictive values, respectively [38, 39]. Sonographic masses that meet criteria for BI-RADS 3, like their counterparts in mammography and MRI, have a less than or equal to 2% likelihood of malignancy. This category reduces the number of false-positive biopsies and justifies a period of watchful waiting, avoiding unnecessary workup when the likelihood of malignancy is very low [20, 40–42].

**Fig. 6:**
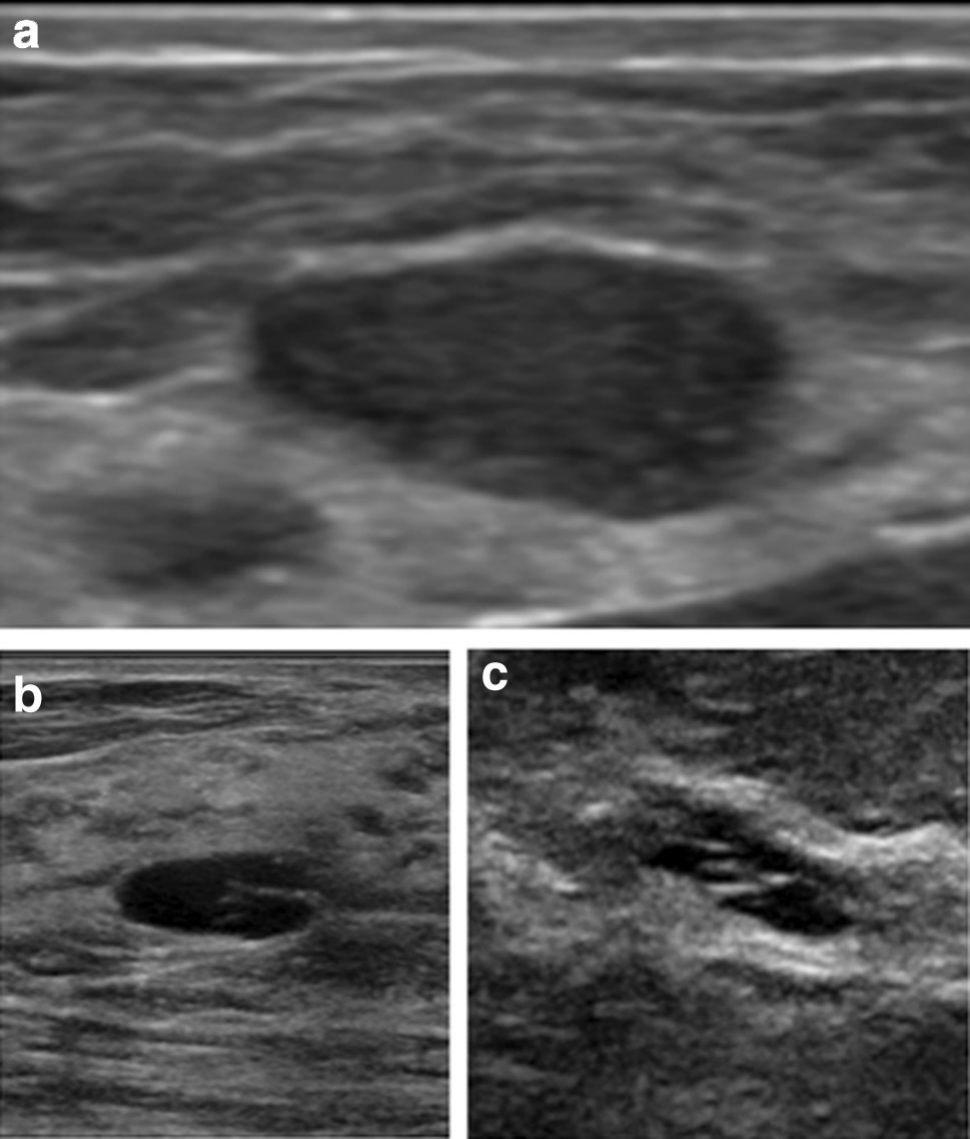
**a** Typical appearance of a BI-RADS 3 oval mass, with circumscribed margins and parallel orientation. **b** Anechoic cyst with thin internal septations, assessed as BI-RADS 3. **c** Clusters of anechoic microcysts, assessed as BI-RADS 3

**Fig. 7:**
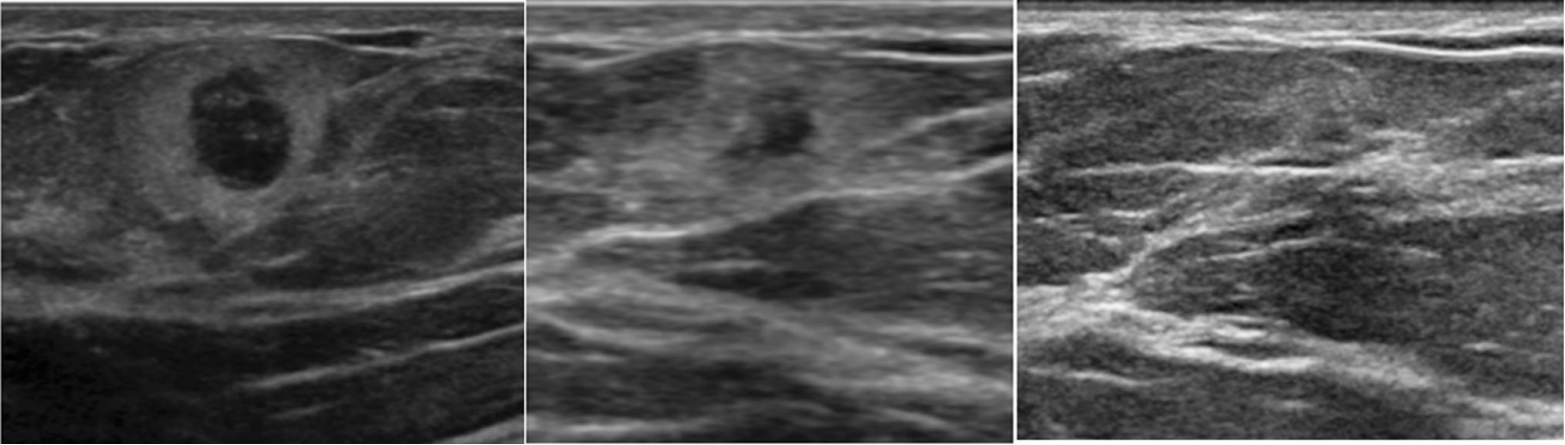
Weeks 0, 6, and 11: suspected fat necrosis in a patient with known breast trauma. Short interval follow-up demonstrates decreasing size of the mass confirming the diagnosis

The characteristics that determine a BI-RADS 3 mass on ultrasound include benign features such as an oval shape, well-circumscribed margins, parallel orientation, echogenicity less than fat with no posterior features or minimal posterior acoustic enhancement [43]. Some masses that are commonly assessed as BI-RADS 3 include classic appearing fibroadenomas, an isolated complicated cyst or cluster of microcysts that is perhaps diagnostically challenging or new in a postmenopausal woman not on hormonal therapy (Fig. 6). Greenwood has shown that no cluster of microcysts was found to be malignant [44], confirming that BI-RADS 2 or 3 are appropriate assessments of the clustered microcysts. There are instances where the personal experience of the radiologist may warrant a shorter interval follow-up, which may not fall into the typical BI-RADS 3 follow-up interval. Such cases include fat necrosis or hematomas, where worrisome imaging appearance may not justify a full 6-month wait time. Commonly, women do not remember trauma to the breast and therefore fat necrosis is often worked up and biopsied. However, when there is known breast trauma and a suspicion of fat necrosis or hematoma, a BI-RADS 3 assessment with a tailored shorter interval follow-up (4–8 weeks) may be considered (Fig. 7). Evolution of the fat necrosis or resolution of a hematoma over a short interval time will confirm the diagnosis. For example, a hematoma will likely transform from a hyperechoic heterogeneous mass to a hypoechoic smaller mass over weeks or months [22••, 39].

BI-RADS 3 is used for both palpable and non-palpable masses and can accurately predict benignity when combining clinical information with mammographic and ultrasound findings [40, 42, 45]. A mass may be categorized as BI-RADS 3 during handheld screening ultrasound—as opposed to screening mammography—as this modality is often read in real time and the findings can be detected and evaluated simultaneously as a combined screening and diagnostic exam. When a woman has multiple bilateral masses, each mass should be evaluated separately and BI-RADS assigned to the most suspicious mass. If all masses are similar and there are more than 3 (with 1 on one side and 2 on the other), data from ACRIN 6666 indicate that they may be assigned BI-RADS 2 [46].

**Fig. 8:**
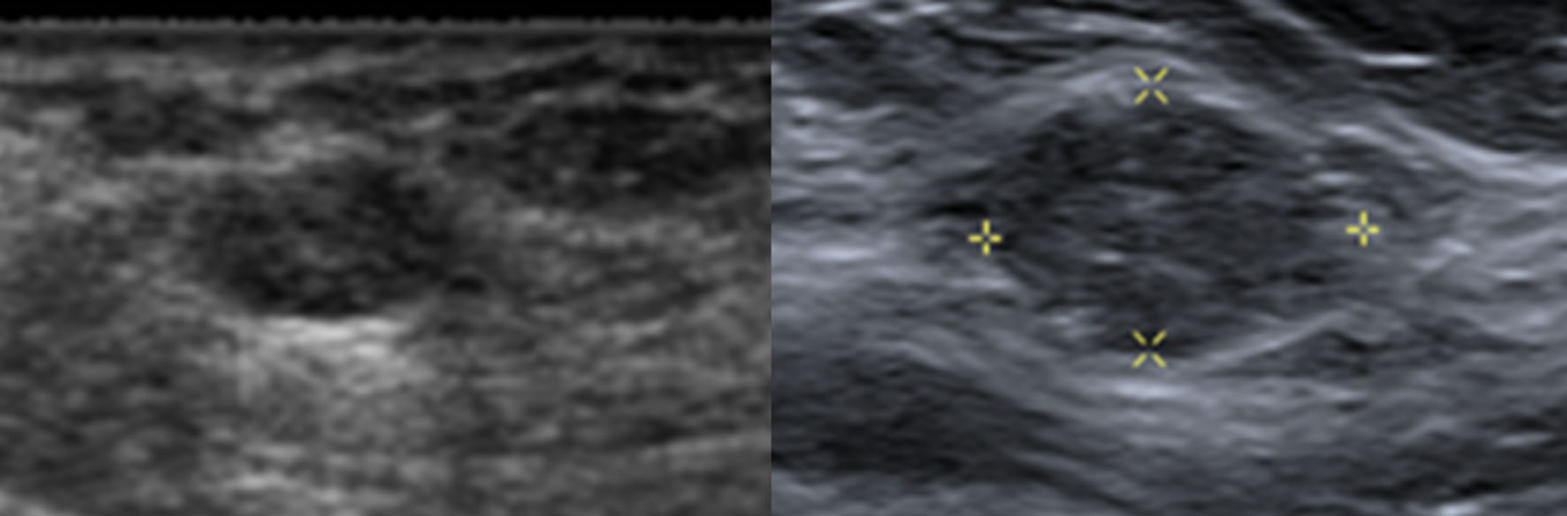
Interval growth of a BI-RADS 3 mass. This solid oval mass on ultrasound was assessed as BI-RADS 3. However, patient was lost to follow up and did not return for 2 years. The mass was biopsied at 2-year follow-up due to interval growth yielding mucinous carcinoma

Similar to the mammographic BI-RADS 3 protocols, ultrasound BI-RADS 3 masses will typically undergo a 6-, 12-, and 24-month surveillance protocol to ensure stability and continued benign appearance. After 24 months of stability, the patient may return to routine screening. If during this surveillance period, the mass decreases in size or demonstrates resolution, it can be downgraded to a BI-RADS 2. If during the surveillance period the mass grows in size or demonstrates suspicious qualities, then the BI-RADS category may be upgraded to a biopsy recommendation. Significant interval increase in size of a mass, generally accepted as greater than 20%, can supersede benign morphology of a mass and often warrants a biopsy [47]. Significant interval growth is concerning for pathologies such as mucinous, medullary, or papillary carcinomas (Fig. 8). A recent study by Jang et al. showed that the malignancy rate for enlarging or morphologically changed lesions was significantly higher than for stable lesions [48]. If the mass is indeterminate or has any suspicious characteristics, a BI-RADS 3 category should not be issued and biopsy should be pursued. In these cases, a wait period is not justified and may delay diagnosis of cancer.

Chae et al. demonstrated the radiologist’s difficulty using BI-RADS 3 for breast ultrasound. In their experience, 14.6% of screening breast ultrasounds were read as BI-RADS 3. When, however, they reinterpreted the ultrasound exams using ACRIN 6666 criteria, 19.3% of cases had an assessment change. Of 225 patients who had a BI-RADS change, 213 were changed to BI-RADS 2 and 12 were upgraded to BI-RADS 4 [35•]. Chae also found that the malignancy rate was higher for those with abnormal mammograms compared with those who had normal mammograms (2.2% vs 0.4%) [35•]. This suggests an avenue for future research.

As ultrasound techniques continue to improve with higher-frequency linear transducers that increase spatial resolution, spatial compounding to improve margin analysis, tissue harmonic imaging that reduces near-field artifacts and intensifies posterior acoustic features, and more robust power Doppler, radiologists have the opportunity to increase their diagnostic confidence [44]. This may lead to further refinements of BI-RADS 3 ultrasound criteria.

### BI-RADS 3 in Breast MRI

BI-RADS 3 for MRI was adapted from mammography [2]. While there are well-established criteria for the use of BI-RADS category 3 for mammography, similar criteria have not been established for MRI. Several studies have demonstrated that lesions assigned to BI-RADS 3 category have a ≤ 2% malignancy rate. Association with specific BI-RADS lesion descriptors, however, could not be established [49, 50]. The majority of studies on the frequency of BI-RADS 3 report that between 6 and 12% of examinations are assessed as BI-RADS 3 (Table 1).

**Table 1:** Frequency of MR imaging BI-RADS 3 assessment category and cancer yield

Reference	Type	Study population	Probably benign examinations (number [%])	Probably benign patients (number [%])	Cancer yield (number [%])
Kuhl et al. [73]	Prospective	High risk	45/363 (12.4)	44/192 (22.9)	1/44 (2.3)
Liberman et al. [74]	Retrospective	High risk	89/367 (24.2)	89/367 (24.2)	9/89 (10.1)
Kriege et al. [75]	Prospective	High risk	275/4169 (6.6)	NR/1909	3/275 (1.1)
Hartman et al. [76]	Prospective	High risk	19/75 (25)	14/41 (34.1)	0/14 (0.0)
Sadowski and Kelcz [77]	Retrospective	BI-RADS 0 mammogram	NR	79/473 (16.7)	4/68 (6)
Kuhl et al. [78]	Prospective	High risk	167/1452 (11.5)	NR/529	NR
Eby et al. [79]	Retrospective	High risk, extent of disease, problem solving	160/809 (20)	160/678 (23.6)	1/160 (0.6)
Eby et al. [59]	Retrospective	High risk, extent of disease, problem solving	260/2569 (10.1)	236/1735 (13.6)	2/236 (0.9)
Weinstein et al. [80]	Prospective	Known contralateral cancer	106/969 (10.9)	106/969 (10.9)	1/106 (0.9)
Hauth et al. [81]	Retrospective	High risk, extent of disease, problem solving	44/698 (6.3)	44/698 (6.3)	1/56 (1.8)
Mahoney et al., 2012 [50]	Prospective	Known contralateral cancer	106/969 (10.9)	106/969 (10.9)	1/101 (0.9)
Lourenco et al. [82]	Retrospective	High risk, abnormal imaging, extent of disease, clinical symptom	348/4370 (8)	NR/345	5/345 (1.4)
Bahrs et al. [58]	Retrospective	High risk, extent of disease, problem solving	182/666 (27.3)	117/NR (17.6)	3/163 (1.8)
Spick et al. [54]	Retrospective	Not high risk, no history of breast cancer	108/1265 (8.5)	108/1265 (8.5)	1/108 (0.9)
Grimm et al. [83••]	Retrospective	High risk, extent of disease, problem solving, clinical symptoms	282/4279 (6.6)	265/3131 (8.4)	12/280 (4.3)
Chikarmane et al. [84]	Retrospective	High risk, diagnostic purposes	NR/5778	483/3360 (14.3)	11/435 (2.5)

*NR* not reported

There are unique features to MRI BI-RADS 3 assessments. The population undergoing screening MRI is at higher lifetime risk for developing breast cancer than those undergoing screening mammography. Also, the clinical indication for performing MRI is different from mammography. Patients undergoing MRI because of a known breast cancer to evaluate the extent of disease have a higher frequency of additional areas of cancer, thus raising the suspicion of findings that would otherwise be less worrisome [51, 52, 53••]. Furthermore, the utility of short-term follow-up for a patient who is about to begin breast cancer treatment is disputable. In addition to the actual finding characteristics, a patient’s breast cancer risk and planned breast cancer treatment should be collectively assessed when MRI findings are assigned BI-RADS 3.

**Fig. 9:**
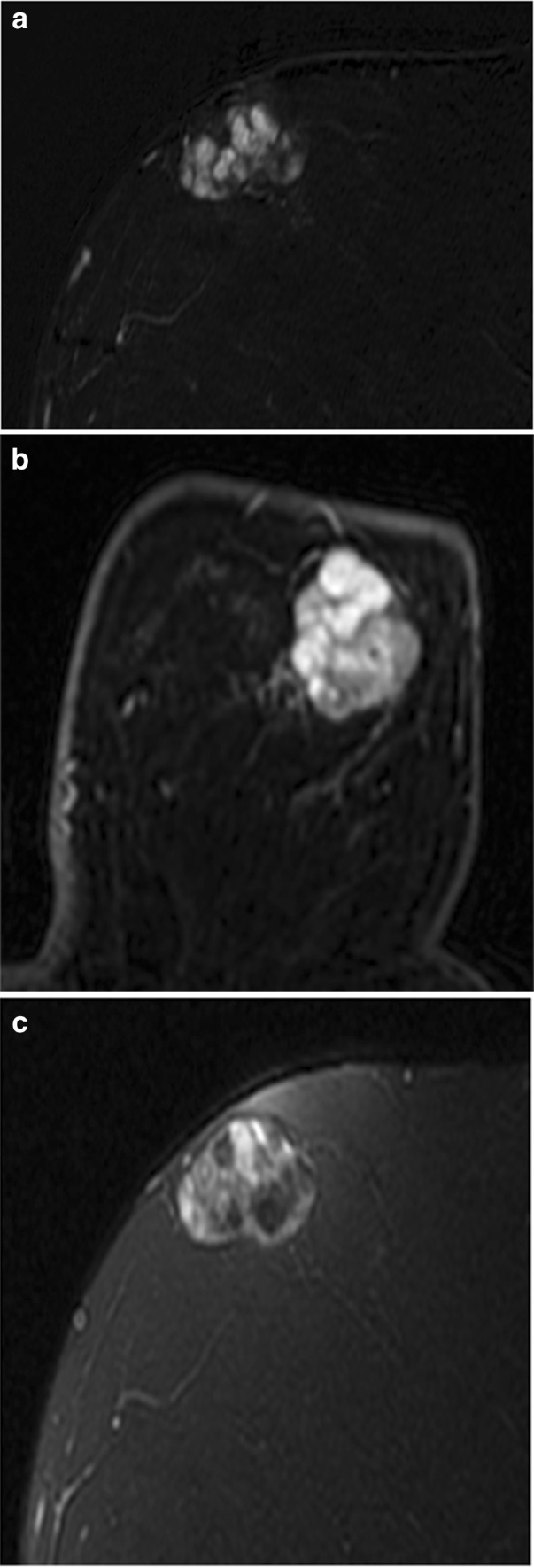
Fibroadenoma. Postcontrast subtraction T1-weighted sagittal (**a**) and axial (**b**) images show a 3.6-cm oval mass with circumscribed margins and dark internal septations. On **c** fat-saturated T2-weighted image, it demonstrated high signal intensity and is most consistent with a fibroadenoma. If this mass was an incidental finding on baseline MRI, a BI-RADS 3 assessment would be appropriate

**Fig. 10:**
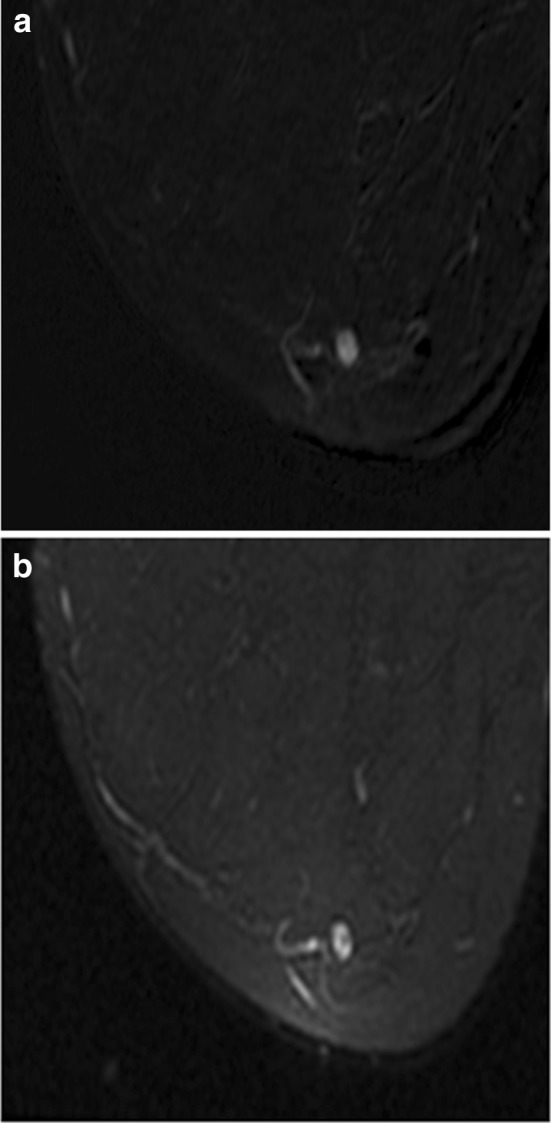
Mass with oval shape and circumscribed margins. **a** Postcontrast subtraction T1-weighted image shows a 0.6-cm oval-shaped mass with circumscribed margins and homogeneous internal enhancement, which demonstrated high signal on T2-weighted sequence (**b**) and a BI-RAD 3 assessment was given. 6-month follow-up MRI showed that this mass was stable and is likely an intramammary lymph node. This example shows that BI-RADS 3 assessment is appropriate for masses with an oval shape and circumscribed margins on baseline examination

A mass is a space-occupying three-dimensional lesion, which has a defined shape, margin, and internal enhancement characteristics. The most appropriate and common use of BI-RADS 3 assessment is for a round- or oval-shaped mass with circumscribed margins and hyperintense T2 signal, which has either homogeneous enhancement or dark internal septations on a baseline examination. A mass meeting these criteria is most likely an intramammary lymph node or fibroadenoma. (Fig. 9). Therefore, a mass with a round or oval shape, circumscribed margins, and persistent or plateau kinetic curve should be assigned BI-RADS 3 on baseline examination [54] (Fig. 10). Although increased T2 signal is most often associated with benign masses, it has been reported in subsets of breast cancers, particularly of the mucinous and papillary subtype [55, 56]. When mass features are studied, the single most predictive feature for malignancy is the margin [25, 57] not the T2 signal.

**Fig. 11:**
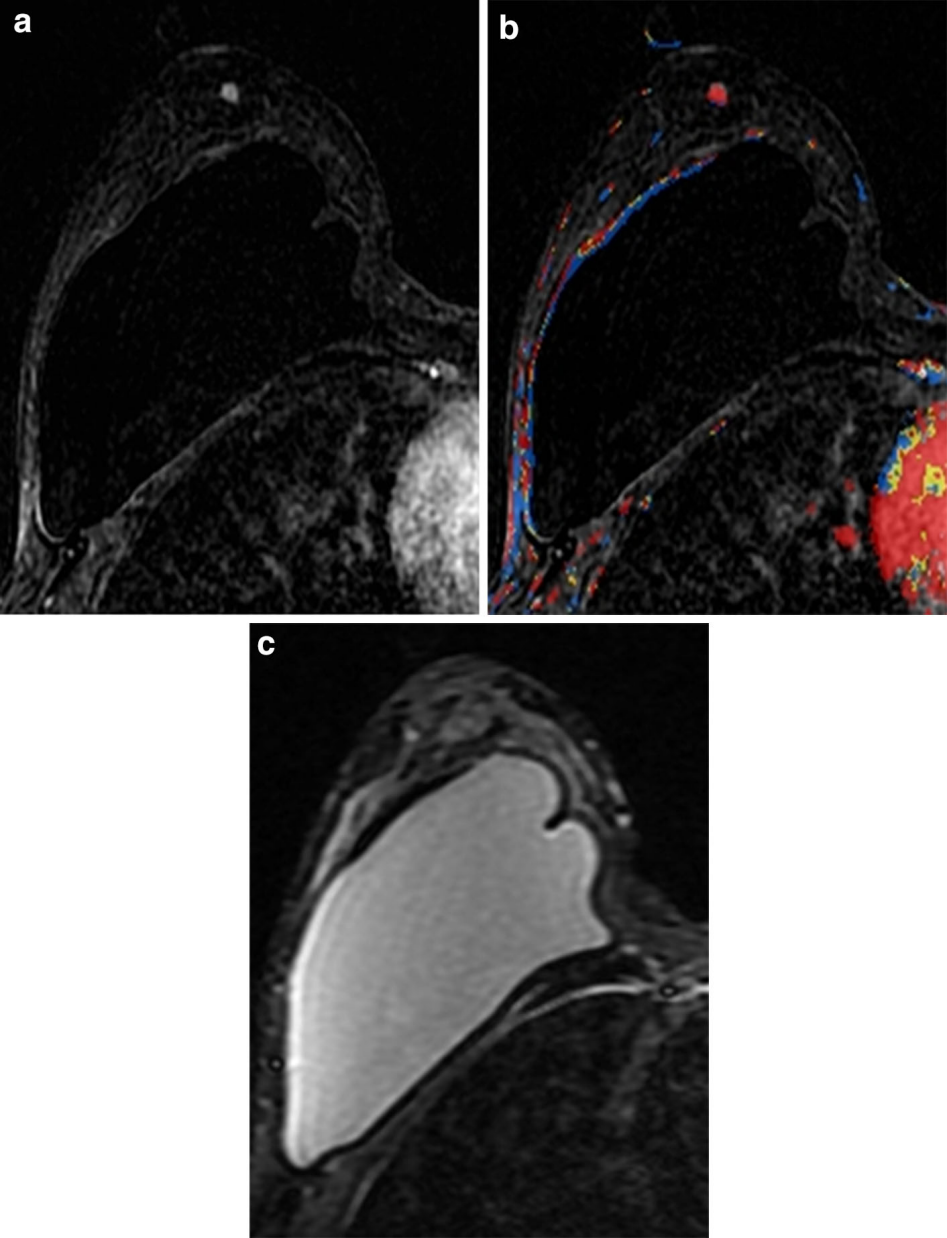
Focus with the absence of high signal on T2 sequence. **a** Postcontrast subtraction T1-weighted image shows a unique 0.4-cm focus with washout delayed kinetics (**b**) and the absence of high signal on fat-saturated T2-weighted image (**c**). Because this focus was new, it was assessed as probably benign, BI-RADS 3. Follow-up examination 6 months later showed increase in size of the focus; therefore, biopsy was recommended. MRI-guided wire localization was performed of this focus and surgery yielded invasive ductal carcinoma. For foci with washout kinetics and the absence of high T2 signal, biopsy should be considered

Foci represent up to 41–48% of BI-RADS 3 lesions [58, 59], but are rarely malignant. A focus is a unique enhancing dot, usually less than 5 mm, which is too small to further characterize. Although the vast majority of foci are benign, new or enlarging foci should raise suspicion and prompt either short-term follow-up or biopsy [58] (Fig. 11).

A study by Eby et al. found that all foci that demonstrated persistent kinetics on delayed phase enhancement were benign, suggesting that all persistent foci can appropriately be assigned BI-RADS 2 [59]. There is, however, conflicting data on the utility of kinetic information in assessing foci. A retrospective study of 111 patients with 136 foci by Ha et al. showed that kinetics were not useful in distinguishing benign from malignant foci [60]. Ha et al. demonstrated a malignancy rate of 2.9% (4 of 136 foci); the predictors of malignancy were an absence of high T2 signal intensity and a focus that was either new or increased in size.

There are limited data to support the use of BI-RADS 3 for non-mass enhancement (NME).

Non-mass enhancement is defined as enhancement that is not a mass and whose internal enhancement characteristics are unique from background parenchymal enhancement (BPE). A study by Schnall et al. showed that distribution was the most predictive of diagnosis in NME [57]. Additional studies have demonstrated that NME with a linear or segmental distribution requires biopsy because these descriptors not only have a greater than 2% malignancy rate but were most predictive of malignancy [50, 61].

**Fig. 12:**
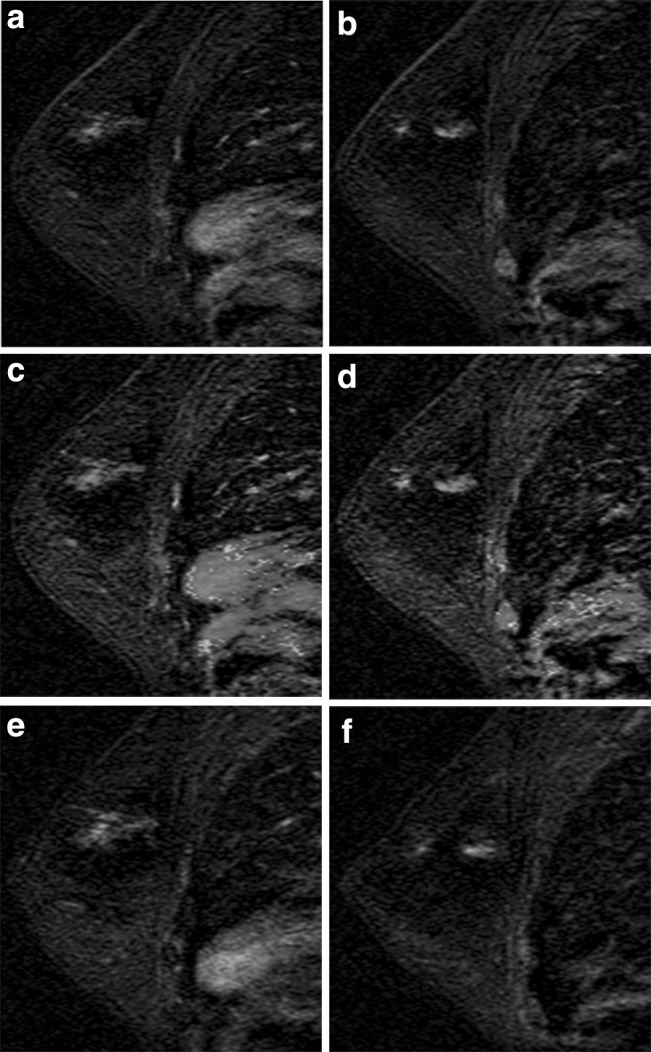
Multiple regions of non-mass enhancement (NME) in the same breast. Postcontrast subtraction T1-weighted images **a**, **b** show multiple regions of NME, which are new but demonstrated persistent kinetics (**c**, **d**). These were assessed as probably benign, given the multiplicity, and were assumed to be transient enhancement related to hormonal status in this premenopausal woman. Follow-up exam 6 months later demonstrates slight increase in degree of enhancement (**e**, **f**); therefore, MRI-guided biopsy was recommended and yielded ductal carcinoma in situ (DCIS). Patient elected mastectomy yielding diffuse DCIS, no invasive component. New areas of NME should raise suspicion and biopsy should be considered

Spick et al. report that BI-RADS 3 may be assigned if the NME is either focal or regional in distribution and the internal enhancement pattern is homogeneous with either persistent or plateau enhancement kinetic curve [54]. Regional, multiple regions, and diffuse distribution patterns were associated with the lowest probability of cancer [50]. It would therefore be acceptable to use BI-RADS 3 for NME with a focal or regional distribution, homogeneous internal enhancement on a baseline examination. However, if there is new focal or regional distribution of NME, suspicion should be raised and a biopsy would be appropriate (Fig. 12).

**Fig. 13:**
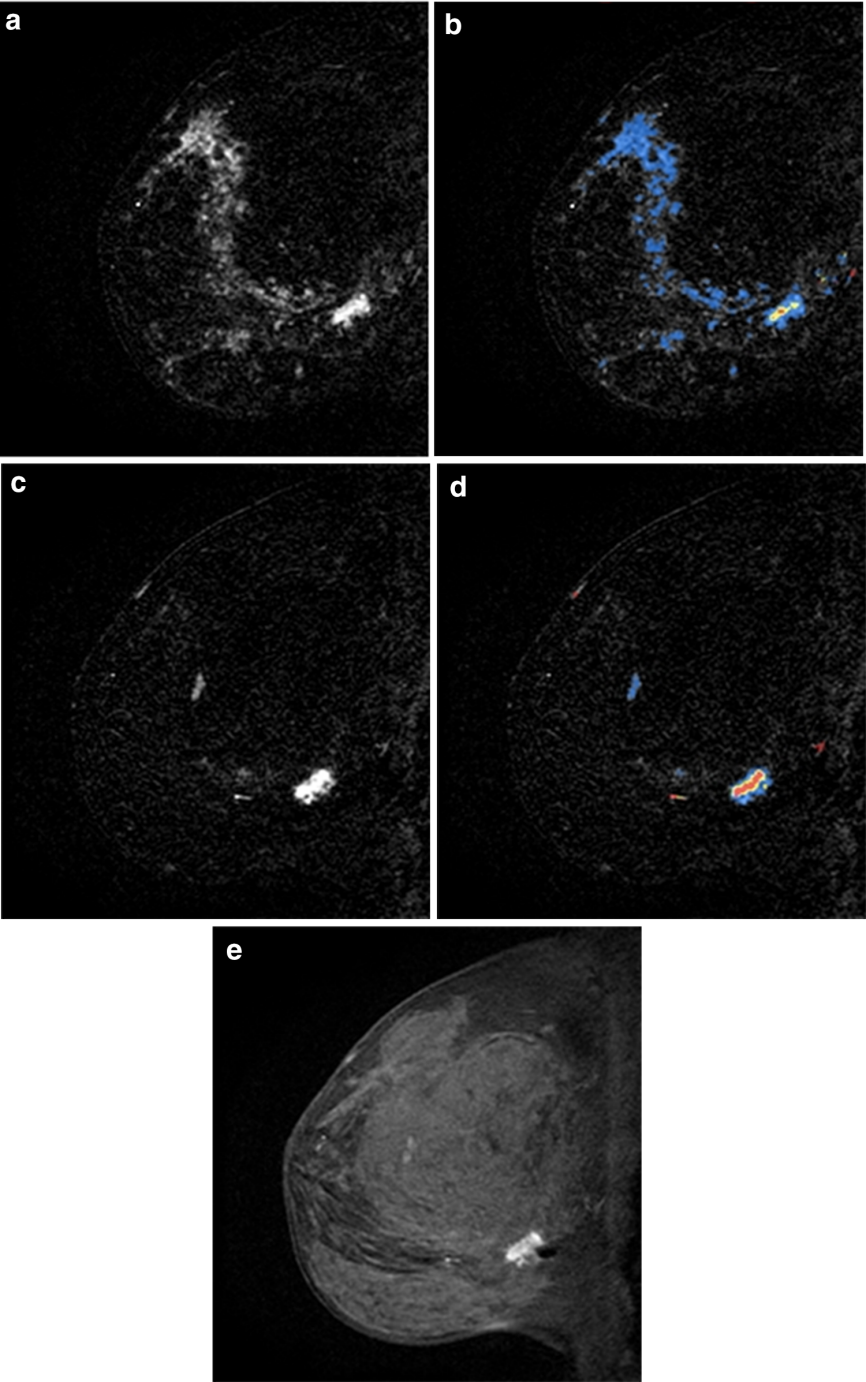
Missed MRI-guided biopsy with follow-up demonstrating cancer. **a** Postcontrast subtraction T1-weighted image shows a 1.2-cm non-mass enhancement (NME) with focal distribution, heterogeneous internal enhancement, and **b** washout kinetics (arrow), which was suspicious and assessed as BI-RADS 4. MRI-guided biopsy was performed yielding fibrocystic changes and a 6-month follow-up MRI was recommended. At 6-month follow-up, **c** postcontrast subtraction T1-weighted image shows persistence of the NME and washout kinetics (**d**). Postcontrast T1-weighted image (**e**) shows that the susceptibility artifact from the biopsy marker clip is located posterior to the focal NME, which was unchanged in size and appearance suggesting that the NME was not biopsied. Surgical excision yielded carcinoma in situ

The 5th edition of the BI-RADS Atlas recommends an MRI short-term follow-up interval identical to that recommended for mammography [2]. Unique to MRI are issues concerning the patient who had an MRI-guided biopsy yielding benign concordant pathology. In this setting, a short-term follow-up MRI is appropriate in order to confirm adequate sampling of the targeted lesion. It is suggested that the most effective timing to perform this follow-up is 6 months after the biopsy [62]. MRI after benign concordant MRI-guided biopsy has shown that 8–12% of targeted lesions were inadequately sampled and, of those inadequately sampled, malignancy was ultimately diagnosed in 14–18% with a false-negative rate of MRI-guided biopsy of 2.5% [63]. Although no study has addressed, the significance of lesion stability 6 months following MRI-guided biopsy, the possibility of a missed target should be entertained (Fig. 13). Cancers, which were missed on MRI-guided biopsy, usually do not demonstrate appreciable change in size sooner than 6 months [64].

While there are no established criteria for the use of BI-RADS 3 for breast MRI, there is increasing experience determining which lesions would be appropriate for BI-RADS 3. An incidental round- or oval-shaped mass with circumscribed margins and hyperintense T2 signal, which has either homogeneous enhancement or dark internal septations on a baseline examination is an appropriate use of BI-RADS 3. It also would be reasonable to assign a focus with washout kinetics into the BI-RADS 3 category on baseline examination. Short-term follow-up can be recommended for homogeneous NME with a focal, regional or multiple regions distribution on baseline examination. When determining whether a BI-RADS 3 assessment would be appropriate, it is imperative to consider the patient’s breast cancer risk and potential planned breast cancer treatment.

### Future Directions in the BI-RADS 3 Assessment Category

Just as the clinical setting is important in MRI, work by Burnside et al. has shown that there are risk factors that should give one pause before assigning a BI-RADS 3. A logistic regression model that included age, personal breast cancer history, family breast cancer history, breast density, and mammogram features was applied to almost 5000 mammograms that had been interpreted as BI-RADS 3. A greater than 2% diagnosis of malignancy occurred in those patients who had a personal history of breast cancer and were over 50 years old who were placed into the BI-RADS 3 category at diagnostic mammography [65]. This work highlights the importance of looking beyond the images before deeming a finding probably benign.

Linda et al. attempted to decrease the numbers of BI-RADS 3 assessments of calcifications by adding a contemporaneous MRI. They sought to determine if a normal MRI would indicate that the BI-RADS 3 calcifications were indeed benign and the patient could be returned to annual screening. Unfortunately, there was no statistically significant difference in the ultimate malignancy rate of those with positive and negative MRI exams. Thus, MRI cannot be used to exclude malignancy in the case of BI-RADS 3 calcifications [66].

Elastography was evaluated by Cho et al. to determine its ability to upgrade or downgrade BI-RADS 3 masses. In their study, 276 BI-RADS 3 masses were evaluated with elastography. No invasive cancers were included in this cohort. 166 had negative elastograms with 1 of those patients having DCIS. If the negative elastograms were used to change the assessment from BI-RADS 3 to BI-RADS 2, in this cohort the malignancy rate would have changed from 1 to 1.8% [67]. Larger studies that include some invasive cancers in the BI-RADS 3 group are needed to validate these results.

Future directions in MRI that have been studied include the use of diffusion weighted imaging (DWI) to determine its utility in the BI-RADS 3 mass. When Dijkstra et al. added DWI with intravoxel incoherent motion to standard MRI, the specificity increased from 30.4 to 56.6% with a negative predictive value of 92.9%. This study is limited by including only large lesions, a very specific technique and small sample size, but points to the possibility of further technical refinements in MRI that could impact patient care [68].

BI-RADS 3 will continue to evolve as we more to making its use ever more evidence based and less intuitive [69]. Continued research is needed to allow the practicing radiologist to properly and consistently use BI-RADS 3 across all breast imaging modalities including the less commonly available contrast-enhanced mammography [70] and molecular breast imaging [71, 72].

## Conclusion

BI-RADS 3, probably benign, is a challenging assessment category. While its use in MRI is evolving, there are specific criteria for the designation of a mammographic or ultrasound finding as BI-RADS 3. Additionally, one’s personal experience may allow other findings to fall into the probably benign category. Using the criteria outlined in the BI-RADS atlas and careful attention to patient characteristics such as age and ability and/or willingness to return for multiple follow-up visits will optimize the use of this most taxing and dynamic BI-RADS assessment category.
